# Massachusetts Veterinary Association

**Published:** 1894-02

**Authors:** John M. Parker

**Affiliations:** Secretary


### SOCIETY PROCEEDINGS.

### MASSACHUSETTS VETERINARY ASSOCIATION.

*October, 1893.*

The regular monthly meeting of the Massachusetts Veterinary Association was held in 19 Boylston Place, on Wednesday evening, October 25th, at 7 30 P. M.

Dr. Burr being absent, the Vice-President, Dr. Winslow, occupied the chair.

The members present were Drs. Blackwood, Ferguson, Howard, Lee, Marshall, Parker, Soule and Winslow. After the minutes of last meeting were read,

*Dr. Blackwood* moved that they be accepted as read. Seconded by Dr. Howard. Carried.

*Dr. Marshall* wanted to hear from some of the members present on the subject of advertising. Dr. Becket had promised at last meeting to have some objectionable matter rectified.

*Dr. Howard* wanted to hear an expression of opinion from Dr. Lee on the matter.

*Dr. Lee* does not know that he has anything to say on the matter. Dr. Becket, of course, has his own opinion and can speak for himself. He is sorry if the Society does not believe in what he had done; he could not change his opinion, however. He further said the veterinary profession had been compared frequently with the medical profession. Personally he does not think they are to be compared. In most cases in veterinary practice it is merely a matter of dollars and cents, in the same way as the practice of law or banking or plumbing or the selling of groceries is merely a matter of dollars and cents. He does not see that there is any practical difference between the veterinary profession and these other money making schemes.

*Dr. Marshall* is surprised that Dr. Lee looks upon the veterinary profession as a trade and not as a profession. It would put a stop to all advancement if we all believed as he did.

*Dr. Howard* thinks Dr. Lee's statement is different from what we are used to. It is not usual to consider it only equal to a trade such as selling groceries or plumbing. Many have worked years to benefit the profession, and we have all benefited by it financially and stand better in the community in every way in consequence. Article I in the Constitution says that one of the objects of the Association is "To use its influence for the advancement of the profession both as a body and as individuals," etc. If our Association has a constitution, why not live up to it?

*Dr. Rogers* does not think any member will agree with Dr. Lee. He must know what it would mean to all of us if the profession was reduced to the level of dollars and cents. He is really surprised at Dr. Lee. Others, and even Dr. Lee himself, have striven to elevate the profession; he does not think Dr. Lee means all he says. Dr. Lee should have time to reconsider the position he has taken.

*Dr. Lee* repeated that he had meant all he had said. Veterinary practice was not the same as medical practice; it could not be compared to it: it was entirely a business transaction.

*Dr. Ferguson* thinks there is a higher plane than any so far expressed; when it is considered merely as a matter of dollars and cents it lowers the profession; we should try to elevate it, not lower it. He considered the rise of the profession in Cambridge a good illustration of the rise of the profession in the country.

*Dr. Parker* thought that the two professions were very closely allied, and the closer they were brought the better it would be for both. The medical and veterinary professions are inseparably connected with each other, and the bonds are growing closer all the time. We all know that many diseases are intercommunicable, and in many cases the medical profession have to call on the veterinarian to aid him. If the profession is reduced to a trade, if it is made merely a matter of dollars and cents, it will ruin the profession; it will have fifty years' work to do over again, and *the highest and best of the profession would be lost;* if everyone looked at it in that light we would have *no scientists;* we would have *no advance;* we would be at *a standstill.*

*Dr. Lee* did not think the profession should be surrounded by sentimental ideas any more than the business of a banker, lawyer, analytical chemist, or a sanitary plumber. He did not mean to lower it, but other businesses besides it have done good to the people in general. The discussion began by criticisms on business methods; there was a popular prejudice against innovations of any kind. The man who introduces any innovation meets with opposition. "In other businesses they are not hampered with codes, and he feels he should not be dictated to as to how he should conduct his business."

*Dr. Blackwood* says, if Dr. Lee wishes to compare it with other professions, let him do so and he will find that even the blacksmiths have their code; if he does not believe it, let him join the union and see.

*Dr. Howard* said that veterinary practice cannot be compared with business, because the science is not a *positive* one. If a person is driving along the street and his wheels get caught in the car tracks, and he has a smash up, he can go to a blacksmith, who will tell him how long, and what it will cost, to have it repaired. On the other hand, if his horse goes lame it is often hard to tell where the lameness is, not to speak of the cost of repair; he may have to call in some brother practitioner in consultation. What would be the result without "Ethics"? Dr. Lee had compared the veterinary profession with plumbers; who drove plumbers into sanitary science but the medical profession? Then he compares our profession with chemists. Who ever saw a prominent chemist putting out advertisements, saying he would do business for so much? It is the same with prominent lawyers and theologians. All professions have either a printed code or an implied code; most of us do not need a code to go by; we have an instinctive knowledge of what is right and fair.

Continuing, Dr. Howard said: "We don't dictate when it comes to the actual practice of the profession; we don't dictate as to the medical or surgical treatment of cases; we only ask that a member should comport himself reasonably to other members of the profession.

*Dr. Lee:* In those trade associations where members have to work so many hours a day, a man is fined when he disobeys. In the veterinary profession if every visit should be charged so much, it would be a great benefit, but as things are now charges are not adhered to; bills are lumped and contracts made, and until the profession advances to the point of having a schedule of prices, until some such arrangement comes, he does not see why he should be charged with being dishonest.

*Dr. Howard:* Dr. Lee says, "Until we have advanced to the point of having a schedule of prices." That is not advancing; who can say I should not give my services for nothing, as a charity? It is not advancing; it is going back, to have a schedule of charges.

*Dr. Ferguson* is glad the subject of contracts has been brought up; when he first went to Cambridge travelling quacks were selling their medicines to the large stables; he did not know how to get round them. After a good deal of consideration he went to the large stables and got a contract with them; they to furnish medicine and pharmacy; he gets all the way from $4.50 to $7 00 a horse.

*Dr. Rogers* thinks contracts make it much harder for young men to get started; he knows where he might have got work if it had not been for contracts.

*Dr. Howard* thinks that is a good point; he does not see how a young man is to get work against contract work; he asks Dr. Ferguson if he would be willing to give up contracts when fakirs were frozen out. Older men should not be jealous if young men are called.

*Dr. Rogers* thinks young men can often underbid older men; he has had chances to do so, but had not done so because he had not thought it right.

The meeting then adjourned.

JOHN M. PARKER, *Secretary.*

*November, 1893.*

The regular monthly meeting of the Massachusetts Veterinary Association was held at 19 Boylston Place on Wednesday, November 22, 1893, at seven-thirty P. M.

The President, *Dr. Burr*, in the chair.

The members present were Drs. Becket, Blackwood, Bryden, Burr, Emerson, Towle, Howard, LaBaw, Marshal, Osgood, Parker, Rogers, Simpson, Winchester and Dr. Stickney, honorary member.

The minutes of the previous meeting having been read and approved, the chair called for the report of delegates to the Chicago meeting.

*Dr. Emerson* reported that the attendance was large and the papers good.

*Dr. Winchester* said that delegates from France and Australia were present. Massachusetts was well represented, as were other States. Some of the papers and discussions were of the greatest interest. The matter of advertising had also been taken up, and was at present receiving the attention of Dr. Hoskins. Instead of the present illumination the seal of the association will be used for letter heads and envelopes ; this change will not be made until the association becomes incorporated, when the seals will be distributed for the use of the members.

*Dr. Becket* said that Dr. Winchester had pretty well gone over the ground. There were some good papers on Tuberculosis, especially one read by Dr. Clement, of Baltimore, who gave an exhaustive resume of experiments; another by Dr. Pearson, of Philadelphia, who discussed the action of tuberculin. In speaking of the specific reaction, he said that in some animals there was no specific rise in temperature from the tuberculin ; these were mostly cases where the disease was well advanced, and in these cases so much tuberculin was already in the system, as a result of the disease, that the few grains introduced did not create any particular disturbance; on the other hand, in the cases not so far advanced, the specific reaction was pretty constant

Remarks were also made by Drs. Bryden and Osgood, both of whom had been greatly impressed by their visit to the "White City."

*Dr. Howard* then moved that the report be accepted and the committee discharged with thanks.

*Dr. Marshal* seconded the motion, which was carried unanimously.

New Business. The secretary then asked the members to volunteer for papers for the ensuing meetings.

*Dr. Simpson* offered a paper on "Digitalis" for the January meeting.

*Dr. Osgood* moved that Dr. W. M. Simpson be asked to contribute a paper at the February meeting. Seconded by Dr. Blackwood. Carried.

*Dr. Charles Simpson* then asked for information as to whether cities are forced to appoint veterinary surgeons as inspectors. In Somerville an inspector had been appointed who was not a veterinary surgeon.

*Dr. Osgood* explained that if an appointee was unsatisfactory to the Board of Cattle Commissioners they could remove and appoint any one they wished at a salary of not over $500.

*Dr. Howard* then rose and said that at the last two meetings the subject of Ethics had been thoroughly discussed; a great deal had been said on the subject, and he would now like to offer a resolution.

*Dr. Rogers* thought it would have to lay over till next meeting to allow due notice to be given.

*Dr. Howard* wanted to know from the President if it was not in order to consider it at the present meeting.

The chair decided that it was in order and could be offered at this meeting.

*Dr. Howard* then rose and said, the question of ethics had been brought up for discussion and certain members had been severely criticised. The association had so far taken no action on the matter, and so he brought forward the resolution to express his feelings on the matter, and to enable the association to put itself on record on the stand taken by Dr. Lee.

At last meeting advertising was fully discussed, and attention was particularly called to one particular advertisement in the press. At that time Dr. Becket said that that advertisement did not receive his sanction. The other day he noticed the advertisement referred to was still in the paper. Consequently he introduced the resolution and sees no reason why it should not be presented.

*Dr. Becket* then said in reference to the last remark of Dr. Howard's he regrets it, and admits he has been negligent in not attending to the matter. It shall, however, be attended to at once. He does not sanction it.

*Dr. Howard* is pleased to hear what Dr. Becket says, as it practically sanctions the gist of the resolution, which is as follows:

WHEREAS, The question of Ethics having received a full discussion, and in that discussion the methods of advertising of certain members having been severely criticised, be it therefore

*Resolved*, That this association hereby express its censure of the promiscuous advertising of the Boston Veterinary Hospital, and commend such management to a sincere consideration of the proper observance of our code.

*Dr. Winchester* moved that the resolution be accepted. Seconded by Dr. Blackwood.

*Dr. Howard* then pointed out that if the resolution is adopted by the association it puts on record its condemnation of such advertising; on the other hand, if it does not adopt them, it puts itself on record as not condemning such advertising.

After some discussion the chair put the resolution, which was carried.

*Dr. Rogers* then showed an interesting specimen of partial fracture of the sixth cervical vertebræ. The horse had been found in the morning with one of his hind feet caught in his halter rope. He was afterward unable to lift his head more than about eighteen inches from the ground. He was killed two months afterward when the bones were found in the condition shown—viz., fracture of lateral processes.

JOHN M. PARKER, *Secretary.*

*December, 1893.*

The regular monthly meeting of the Massachusetts Veterinary Association was held at 19 Boylston Place, on Wednesday, December 27, 1893, at seven thirty P. M.

The President, Dr. Burr, in the chair.

The members present were Drs. Becket, Bryden, Burr, Carlton, Emerson, Howard, LaBau, Marshal, Osgood, Parker, Rogers, Sherman, Winchester and Winslow.

The minutes of the previous meeting having been read, Dr. Osgood said he wished to make a correction ; in answer to Dr. Simpson's question about the appointment of Inspectors he had said that the original appointment lay with the cities and towns. The Inspector need not be a Veterinary Surgeon. If he was not satisfactory to the Board, however, he could be removed by them, and another Inspector appointed at a salary of not over $500 per annum. With this correction the minutes were adopted.

NEW BUSINESS.

The Secretary then read a letter from the new "Veterinary Magazine" asking the support of the Association.

*Dr. Osgood* moved that the new Journal get copy of the Society proceedings as well as the old Journals ("Veterinary Review" and "Journal of Comp. Med"). Carried.

*Dr. Carlton* then read an interesting paper on "Homeopathy as applied to Vet. Med." In the discussion which followed Dr. Winchester said that he had never "hung up his shingle" as a homeopath, at the same time he is a firm believer in small dosage ; one of the most useful drugs he has used is "Veratrum Vivide" ; he has used it for a number of years for all sthenic troubles, and all sthenic febrile conditions from whatever cause, in small doses, repeated frequently. In speaking of the difference in the action of drugs in different cases he said that he had a 1600 pound horse that morning sick with Azoturea ; he had given a hypodermic injection of 2½ grains morphine and less than one-half a grain atropine at 10 A. M. ; at 4 P. M. the horse was "blind as a bat." At other times he has given as much as 40 grains morphine and 5 grains atrophone, and has not had the same results ; on the other hand he has seen a horse crazy from 1½ grains morphine and ¼ grain atropine.

*Dr. Carlton* thinks the result in these cases was due to the atropine being in excess.

*Dr. Winchester* says it is a fact that we can give some horses proportionally a much greater amount than others.

*Dr. Osgood* thinks Dr. Winchester gave excessive dose in combination ; has never seen horse get 3 grains morphine and 1 grain atrophine without going crazy. The combination intensifies the action of both drugs. In the horse opium has the same action as Belladona.

*Dr. LaBaw* cited a case he had seen in Springfield with Dr. Osgood who had given 1½ grains morphine and ¾ grain atrophine when he, Dr. LaBaw, arrived some time later he found the horse pressing his head against the wall, with dilated pupils.

*Dr. Osgood* believes that in aconite small doses repeated are better than large ones. After some further discussion Dr. Winchester moved that a vote of thanks be given the essayist. Seconded by Dr. Winslow and carried unanimously.

*Dr. Bryden* then read an interesting paper on Quittor. In the disscussion which followed Dr. Bryden said that the average time he allowed for a true Quittor to heal was seven months.

*Dr. Marshal* said that when he was with the St. R. R. Co. he had had a great number of cases of quittor. These cases were treated with sulphate of copper injections and poultices; he had not found it necessary to treat any horse for seven months. They had usually good results.

*Dr. Bryden* said it was not fair to take a number of cases and bunch them; the history in each case should be co1sidered separately. Where tissue changes have gone on it was impossible for them to heal in a few weeks.

*Dr. Howard* asked the essayist if he keeps the horse at work or if he poultices and lays still.

*Dr. Bryden* says that where the horse is not same he poultices and works.

*Dr. Sherman* says his treatment consists in paring away all the horn in the neighborhood of the sore; he puts shoe on and does not touch sinous. Whenever he has probed and injected he has had more trouble. He simply keeps hoof away from shoe and the case gets well.

*Dr. Bryden* explained that he considered true quittor to be where there was a condition of hoof where the tissues are confined and degenerated (not so far as necrosis), but a certain amount of degeneration has gone on and the circulation is dammed back. Then, when by the removal of horn and softening of the foot by poultices, the obstruction to the circulation is removed; the tissues spring back into life again If a bird's plumage is removed it is impossible to make them grow in a day or two In the same way, if a foot is confined, and the coronet tight, the tissues below are interferred with, a process of degeneration goes on, cartilagenous changes set in, and the cartilage hypertrophic.

He thinks that the pathological changes in the foot ought to be thoroughly studied.

*Dr. Winchester* referred to paper read at the meeting in Chicago where the fistulæ was due not to external violence, but to internal causes; he thinks that is what takes place in the form of quittor referred to by Dr. Bryden. The speaker then referred to a case of sidebones he had treated on Dr. Bryden's plan with excellent results.

*Dr. Howard* remarked that they all admitted that quittor could arise as Dr. Bryden described; some of those present seemed to think that it was only one form of quittor; according to Dr. Bryden it is a matter of faulty diagnosis.

After some further discussion the meeting adjourned with a hearty vote of thanks to the essayist.

John M. Parker, *Secretary.*

